# Home-based work and childbearing

**DOI:** 10.1080/00324728.2023.2287510

**Published:** 2024-02-06

**Authors:** Beata Osiewalska, Anna Matysiak, Anna Kurowska

**Affiliations:** 1 University of Warsaw; 2 Cracow University of Economics

**Keywords:** fertility, childbearing, work from home, remote work, telecommuting, work flexibility

## Abstract

We examine the timely yet greatly under-researched interplay between home-based work (HBW) and women’s birth transitions. Past research has shown that HBW may facilitate and/or jeopardize work–family balance, depending on the worker’s family and work circumstances. Following that research, we develop here a theoretical framework on how HBW can facilitate or hinder fertility. Using the UK Household Longitudinal Study 2009–19 and random-effects cloglog regression, we study the link between HBW and first- and second-birth risks. We find that HBW is negatively associated with the transition to motherhood and unrelated to the progression to a second child. We also show that HBW helps to enable women to have children if they would otherwise face a long commute. All in all, our findings do not support the idea that the spread of HBW will lead to an immediate increase in fertility.

## Introduction

Incompatibilities between paid work and care have been identified as a main driver of the dramatic declines in fertility that have been occurring in industrialized countries in the second half of the twentieth century (Brewster and Rindfuss 2000; Engelhardt et al. 2004). These incompatibilities were partly a product of the separation of paid labour from the family sphere during industrial revolutions, when workers began working outside the home (Goldscheider et al. 2015). The digital revolution and the development of information and communication technologies are changing the status quo again and allowing at least some paid work to be brought back into the home. The Covid-19 pandemic has shown that home-based work (HBW) is a realistic option for a substantial number of workers (ILO 2020). Will the opportunity to work from home ease the incompatibilities between paid work and family, thus laying the groundwork for higher fertility?

There is no obvious answer to this question. On the one hand, HBW may improve work–family reconciliation by allowing workers to do away with or cut down on commuting or to organize their work more flexibly around family obligations (Felstead et al. 2002; Chung and Van der Lippe 2020). On the other hand, HBW may exacerbate work–family conflict by blurring the boundaries between paid work and family life (Demerouti et al. 2014) or increasing work intensity (Kelliher and Anderson 2010; Felstead and Henseke 2017). It may also have negative consequences on workers’ careers (Munsch 2016), thereby affecting fertility. While a great deal of research has been done on the various consequences of HBW on workers’ lives, studies examining the links between HBW and childbearing are scarce. Two studies that addressed this topic focused directly on fertility intentions. Sinyavskaya and Billingsley (2015) showed that Russian women with access to HBW reported higher first- and second-birth intentions. Kurowska, Matysiak and Osiewalska (2023) found evidence for an overall negative relationship bewteen HBW and fertility intentions for Polish mothers during the COVID-19 pandemic. Other studies that have mentioned a potential HBW–fertility link have addressed it rather indirectly: for example, by looking at the widening use of broadband internet and its effects on fertility but not in the context of HBW (Billari et al. 2019; Liu et al. 2021).

We contribute to this body of research by examining the interplay between HBW and birth transitions in the pre-pandemic context of the UK. So as to eliminate the confounding effects of the pandemic (e.g. school closures) on HBW and fertility, the pandemic years are not included. We measure HBW as the perceived access to and use of working from home, both on a regular and irregular basis. The UK was chosen because HBW was more prevalent there prior to the pandemic than in many other countries. While HBW was not yet a common mode of working, British parents had been legally entitled to request it—along with other flexible working arrangements—since 2003. This right was implemented with the intention of easing work–family tensions in a country with poor public childcare provision and high pressure on men to work long hours (Adler and Lenz 2015; Yerkes and Javornik 2019). And indeed, the share of employees in the UK who worked from home was among the highest in Europe before the onset of the pandemic, with approximately one in four employees working from home at least occasionally in 2019 (Eurofound 2020; Felstead and Reuschke 2020).

Women are the focus of our enquiry, as in the UK they are still mostly responsible for childcare and thus more likely than men to combine paid work with childcare (McMunn et al. 2020). Women have also repeatedly been found to work from home explicitly to accommodate work and family duties, whereas men work from home to prolong their working hours or avoid workplace interruptions (Sullivan and Lewis 2001; Powell and Craig 2015). In order to provide a comprehensive overview of the interplay between HBW and fertility, we study two outcomes: transitions to first and second births (the most common birth transitions in the UK).

Our study is timely, as HBW will likely be more common in the pandemic’s aftermath than it was prior to the pandemic (Dingel and Neiman 2020). The study also contributes to the demographic literature on paid work and fertility (Brewster and Rindfuss 2000; McDonald 2000); this literature has established numerous factors that ease the conflict between paid and unpaid labour and thus help working women to have children. These factors include public policies (Baizan et al. 2016), working-time flexibility (Begall et al. 2015), and male partners’ involvement in childcare (Torr and Short 2004; Cooke 2009). In this study, we introduce and test the role of another factor: workplace flexibility. We outline theoretical mechanisms through which HBW may affect fertility transitions, and we provide empirical evidence for the pre-pandemic period on whether women who had the opportunity to work from home were more or less likely to have children. This evidence is rather descriptive, as many workers that were potentially able to work from home prior to the pandemic may not have taken the opportunity to do so. Nonetheless, the study has the potential to stimulate future research on the topic covering the post-Covid years, once data become available. Finally, we contribute to the literature on HBW, which enumerates numerous benefits of this working arrangement for workers’ lives, including improved work–family balance (Gajendran and Harrison 2007; Allen et al. 2013), psychological well-being and health (Oakman et al. 2020), time use (Powell and Craig 2015), working conditions (Pabilonia and Vernon 2022), and career success (Golden and Eddleston 2020) but not yet fertility.

## Conceptual framework

### Interdependencies between paid work and childbearing

The interdependencies between childbearing and women’s professional careers are complex. On the one hand, paid work could potentially have a positive impact on birth transitions since it provides income, which is indispensable for family formation (Becker 1991; Oppenheimer 1997). An increasing number of studies have demonstrated that women, particularly the highly educated, tend to postpone the transition to motherhood until they have established a secure position in the labour market (Matysiak 2009; Ní Bhrolcháin and Beaujouan 2012; Wood and Neels 2017). On the other hand, women who work for pay, and especially those on steep career trajectories, experience the opportunity costs of parenting and may delay or even forego motherhood (Gustafsson 2001; Nicoletti and Tanturri 2008). The opportunity costs of having a subsequent child and the tensions a mother experiences while combining paid work with taking care of her first child may also discourage her from having another (Bernhardt 1993; Kravdal 1994; Balbo et al. 2013; Goldscheider et al. 2015). It has been argued and empirically demonstrated that shorter or fewer career breaks and lower tensions between paid work and childrearing both facilitate birth transitions (Ariza et al. 2003; Begall and Mills 2011; Dommermuth et al. 2017).

In this context, HBW has the potential to influence childbearing behaviour by creating new opportunities for women to remain in employment and combine paid work with care. In the remainder of this section, we first discuss how HBW affects the opportunity costs of parenting, women’s work careers, and work–family tensions (in the two following subsections on benefits and drawbacks of HBW), before moving on to outline the possible relationships between HBW and childbearing (in the subsequent three subsections).

### The benefits of home-based work

HBW offers numerous advantages that may allow mothers to return to work faster, thereby reducing the opportunity costs of parenting. Empirical research has shown that mothers who have the opportunity to work from home are more likely to work for pay (Matysiak et al. 2022) and less likely to reduce their working hours after childbirth (Chung and Van Der Horst 2018; Arntz et al. 2019). There are several reasons for this. First, home-based workers can organize work more flexibly around family obligations: for example, working when children are in day care or school, in the care of a babysitter or another person, or sleeping (Felstead et al. 2002; Chung and Van der Lippe 2020). Second, teleworkers also save time by not having to commute. They experience fewer workplace interruptions and can carry out paid work in parallel with some household tasks, such as laundry and cooking (Bailey and Kurland 2002; Hill et al. 2003). Finally, HBW may also enable parents to be more present in their children’s lives, even if the children are sometimes in the care of another person (e.g. babysitter, relative) or are mature enough to manage without direct supervision (Callister and Singley 2004).

### The drawbacks of home-based work

At the same time, HBW may increase work–family conflict and jeopardize women’s professional careers. In the absence of physical boundaries between the workplace and home, and with no clear definition of the beginning or end of the working day (both implicit features of HBW), the boundaries between paid work and family life may blur (Glavin and Schieman 2012; Lott 2020). Indeed, employees working from home often experience interruptions during their work time, multitask more, and tend to prolong their work late into the evening (Hill et al. 2003; Powell and Craig 2015). They may also work harder or longer than their on-site counterparts in order to compensate for their absence from the workplace (Kelliher and Anderson 2010; Felstead and Henseke 2017). There is evidence that women who work from home are expected by other household members to do more housework (Ammons and Markham 2004; Hilbrecht et al. 2008) and experience a higher total workload than women who leave the house to work (Kurowska 2020).

HBW may also diminish women’s opportunities for professional development, despite improving their chances of being employed. Teleworkers tend to have fewer networking opportunities, less influence over what is happening at the workplace, and poorer access to training (Baruch 2000; Martinez and Gómez 2013). They are also less visible at work (Richardson and Kelliher 2015) and may be perceived as less committed to their work, thus they may experience ‘flexibility stigma’, that is, the negative perception of employees who use flexible work arrangements by employers and co-workers (Coltrane et al. 2013). Consistent with these arguments, studies have found that home-based workers are, net of their work effort, less likely to be promoted (Fernandez-Lozano et al. 2020) and tend to earn lower hourly wages (Arntz et al. 2019; Golden and Eddleston 2020).

### Home-based work and childbearing

Given the numerous consequences of HBW, both positive and negative, for women’s work careers and their ability to balance paid work and care, it remains unclear whether HBW will have an overall positive or negative effect on women’s childbearing behaviours. Clearly, the effects may depend on parity, as different aspects of HBW are likely to play a crucial role for childless women vs for mothers. Childless women who work from home, or at least have that option available to them, may be more likely to have children than women who work on-site (or even those with no job). This is because they may perceive HBW as an attractive working arrangement that could allow them to return to paid work more quickly after birth, thereby reducing their opportunity costs. These women do not yet have experience of working from home while taking care of children, and thus they may not be aware of the negative consequences of HBW on work–life balance. However, childless women who work from home may be aware of the negative consequences of this working arrangement for their prospects for promotion. If so, they may postpone motherhood until they become more established in their careers.

The story may be different for women who already have one child. These women, if working from home, are already experiencing both the positive and negative consequences of combining teleworking and childcare. While they may enjoy working for pay, saving time on commuting, and being more present in their children’s lives, their working time may also be more fragmented, and they may multitask more and shoulder greater responsibility for both childcare and housework than women who leave the home to work. Moreover, HBW may limit their opportunities for promotion. It remains to be established, however, whether the negative consequences of HBW on women’s work–life balance outweigh the benefits and whether the perceived risk to their promotion opportunities will lead women to postpone the decision to have a second child as they strive to improve their position on the labour market or, conversely, speed up their transition to second birth in the presence of low opportunity costs.

### Home-based work vs part-time work

There are numerous flexible working arrangements that parents, and mothers in particular, can seek out in order to combine paid work and care. Part-time work is the most common (Messenger 2018; Harkness et al. 2019). Reduced working hours allow mothers to stay active in the labour market while lowering work–family conflict by freeing up time that can be flexibly distributed around family needs (Van Breeschoten and Evertsson 2019). Thus, part-time work is often seen as a win–win strategy for women who want to work and reproduce (Hill et al. 2004). As such, if a mother works part-time, she might be less likely to need any other flexible working arrangement (including HBW) than a full-time working mother, who in all probability faces more serious time constraints. Thus, HBW might help full-time working mothers to have children more than it helps women who work part-time.

### Home-based work and commuting

Women, especially those of childbearing age, strongly prefer shorter commutes (i.e. the distance between where they live and work) (Nafilyan 2019; Petrongolo and Ronchi 2020). They are prepared to postpone career development and incur wage penalties in exchange for spending less time going back and forth between the home and workplace (Le Barbanchon et al. 2020; Skora et al. 2020). Consistent with these findings, other researchers have demonstrated that long commutes substantially increase work–family conflict (Voydanoff 2005; Bai et al. 2021). It is therefore reasonable to expect that for women working on-site, the likelihood of having a(nother) child will fall as commuting time increases. Women with long commutes will likely face high opportunity costs of childbearing, since it will be more difficult for them to return to paid work and to combine it with family responsibilities than it is for women with short commuting times (Harkness et al. 2019; Skora et al. 2020). However, opposite effects might be expected for women who can work from home. If they are able to escape everyday commuting thanks to regularly working from home, then the further they live from their workplaces, the greater the time savings from HBW they will perceive; consequently, the greater the reduction in work–family conflict they perceive, the easier it will be for them to decide to have a child.

In summary, given the complex theoretical relationships between HBW and childbearing, as well as the complete lack of previous empirical studies on the relationship between HBW and fertility, we abstain from formulating specific hypotheses on the expected direction of the overall relationship between working from home and birth transitions. However, we expect that: (1) women who work from home (or have the option of doing so) and who would otherwise face a long commute will be more likely to have a child than women who work from home but live only a short distance from the office; and (2) full-time working mothers will be more likely to have a second child if they are able to work from home than if they do not have that option.

## The UK context

We conducted our study in the UK, where total fertility during the analysed period exhibited a decline from a peak of approximately 1.90 in 2009–12 to 1.63 in 2019 (Berrington et al. 2022; OECD 2023). This downturn in total fertility has been attributed predominantly to a decrease in first-birth rates, further underscoring the UK’s position among those countries experiencing high levels of childlessness (Berrington 2017; Ermisch 2021). At the same time, the employment rate among women in the UK is relatively high both prior to and after childbirth compared with other European countries. In 2019, 82 per cent of childless women and 74 per cent of mothers aged 20–49 in the UK were employed, somewhat higher than the EU-28 averages (74 and 70 per cent, respectively) (Eurostat 2022a). Nonetheless, combining paid work and care in the liberal welfare state context of the UK is rather difficult. Care responsibilities in the UK were for a long time left to families (Lewis and Campbell 2007), and the country’s supply of public childcare is restricted (Yerkes and Javornik 2019). Private childcare, in turn, is very expensive, running to one-third of an average couple’s wage (OECD 2022). Women in Great Britain are entitled to 52 weeks of maternity leave and 18 weeks of parental leave, but only the first six-week period is well paid (at 90 per cent of the woman’s average pre-birth weekly earnings), while the rest is paid either at a flat rate (33 weeks) or unpaid (GOV.UK 2022b; similar applies to Northern Ireland, see NIDIRECT.GOV.UK 2023). Leave entitlements have now been extended to men, but fathers rarely make use of them (Kaufman 2018).

These entitlements aside, women are most likely to have a child when they are not employed (Schmitt 2012; Inanc 2015). Those who work for pay before birth usually switch from full-time to part-time work after they become mothers (McMunn et al. 2020). In the UK in 2019, nearly half of mothers worked part-time, compared with less than 7 per cent of fathers (Eurostat 2022b). In addition, British men work relatively long hours compared with men in other European countries (Cousins and Tang 2004; Eurostat 2022c).

Women in the UK solve their work–life incompatibility problems not only by reducing their working hours but also by pursuing flexible working arrangements, including HBW. Indeed, 26 per cent of women who worked in the UK in 2019 did so at least sometimes from home, a proportion that ranked the UK among the highest in Europe (alongside the Nordic countries) (Eurostat 2022d). It is noteworthy that the UK has been characterized by a relatively high proportion of home working since the late 1990s.

The right to request flexible working, including HBW, is guaranteed by British law (GOV.UK 2022a). Britain’s flexible working policy was introduced in 2003, explicitly to ease work–family tensions and support women’s employment. Initially granted only to parents of children under six, it was gradually extended to all workers who had been employed at their workplace for the previous 26 weeks. Among all available flexible working arrangements in the UK, HBW is the most preferred (Van Wanrooy et al. 2013). It is particularly widespread among white-collar workers in service jobs that do not require frequent face-to-face interaction (e.g. financial, legal, or scientific services) (Felstead and Reuschke 2020). Approximately 45 per cent of workers who held managerial or professional positions in 2019 reported having worked from home, a far greater share than the 5 per cent of those working in elementary occupations (ONS 2020).

## Data and method

### Data

For this study we used the UK Household Longitudinal Study (UKHLS; also known as Understanding Society) Waves 1–10, which cover the period 2009–19 (ISER 2022). The UKHLS interviewed members of approximately 40,000 households in Wave 1, making it one of the largest annual longitudinal studies. It collects information on many aspects of peoples’ lives, including their family and professional careers. It contains questions on both partners’ employment status, the availability and use of HBW, other job characteristics, housing conditions, and involvement in unpaid labour.

### Sample

From the UKHLS data set, we selected women of reproductive age (18–44) living in a heterosexual union. We focused on co-resident partnerships, thus we excluded observations (woman-years) in which a woman was not living with a partner (i.e. was separated, divorced, widowed, or single). We accounted for the possibility of a change of partner during the study period by including woman-years for as long as a woman was in one union and then again after she had entered another union. We initially selected only those women who had participated in at least two UKHLS waves, not necessarily consecutive ones (12,000 women), and whose partner had also been interviewed (leaving 10,000 women). Next, we excluded self-employed women, because their work conditions, including those related to workplace flexibility, differ significantly from those of employed women (leaving us with 9,700 women). We focused on first- and second-birth transitions, as there were too few third or higher-parity births for a reliable analysis to be carried out. We thus distinguished two event-history subgroups (in terms of woman-years): childless women for the transition to a first birth (3,054 women) and first-time mothers for the transition to a second birth (3,248 women). We observed these women either until the birth of a child or, if no birth occurred, until their last participation in the survey or end of their partnership, whichever came first. If a woman entered into another union, she was reintroduced to the sample. Furthermore, if a woman took part in the survey both before and after the birth of her first child, she was included in both the subsample of childless women and the subsample of first-time mothers. We further constrained our sample to women for whom we had an adequate number of observations to lag the explanatory variables with respect to the birth of a child. Lagging required that each woman was observed for at least two consecutive waves (and in some cases even three waves if a woman was pregnant in a wave preceding the birth). This procedure further reduced our sample to 2,442 childless women and 2,542 first-time mothers. Finally, we selected only cases with complete information for the HBW measure and other moderating and control variables (described in the following subsections).

Our final sample consisted of 2,095 childless women (5,703 woman-years) and 2,164 mothers (5,329 woman-years). Within this sample there were 765 first and 893 second births. For later analysis, we further limited these samples to employed women only, in order to check the moderating role of commuting time (1,914 childless women and 1,733 mothers). To check that the performed sample selection did not result in a bias towards specific social groups, we compared the socio-economic characteristics of women included in the initial and final samples, taking into account their age, educational level, occupation, and income. This comparison revealed no substantial differences between the two samples (results available on request).

### HBW measure

Our key explanatory variable in this paper is *perceived access to and use of HBW*. It was constructed on the basis of two survey questions. The first was regarding the availability of flexible arrangements at the workplace (*jbflex7*): *If you personally needed any, which of the following arrangements are available at your workplace?* Respondents were asked to choose all answers that applied to them from a set of answers, one of which was *To work from home on a regular basis*. Those who reported having access to flexible working arrangements were asked the second question (*jbfxuse7*): *Do you currently work in any of these ways?* Again, one of the possible answers was *To work from home on a regular basis*. Based on these two questions and the information about women’s labour market status (*jbstat*), we built our measure of the accessibility and use of HBW. It consists of four categories:

- *On-site working* (reference category): women who cannot work from home on a regular basis; this group accounts for 70.8 per cent of woman-years selected for the analysis of first-birth risk and 64.4 per cent of woman-years for second-birth risk;
- *Irregular HBW*: women who can work from home on a regular basis, but either do not make use of this possibility at all or use it on an irregular basis (13.7 per cent of first-birth and 8.6 per cent of second-birth woman-years);
- *Regular HBW*: women who work from home on a regular basis (6.9 per cent of first-birth and 6.4 per cent of second-birth woman-years);
- *Not employed*: women who are non-employed or inactive (8.6 per cent of first-birth and 20.6 per cent of second-birth woman-years).

Our approach to measuring HBW thus pertains to its accessibility and use on a *regular* basis. It is important to note that regularity in this context does not just imply exclusive work from home but also the ability to combine work performed at the employer’s premises with consistent work from home. This strategy for measuring HBW aligns with the approach applied in past studies based on the UKHLS (Chung and Van Der Horst 2018, 2020). Previous studies have argued that even perceived access to HBW might benefit workers as they may see it as a resource for future use (Kossek et al. 2006). As such, women with access to HBW may plan to make use of their eligibility after childbirth or when their future family or work demands necessitate it. A further group of women have access to HBW but may consider it inconvenient and thus forgo it entirely. Unfortunately, within our category *Irregular HBW*, it was not possible to separate the subcategory of irregular users of HBW from those responding *I have access to HBW but do not use it*.

In addition, because the UKHLS asks respondents about their main job location, those who work mainly from home could be identified. However, very few women (less than 2 per cent of the sample) indicated that they *mainly* worked from home: too few to perform the interactions included in our models. As a sensitivity check, we added this variable to our basic model (see Robustness checks subsection).

### Moderating variables

We tested the role of women’s part-time schedules and commuting time for the relationship between working from home and birth risks. We did this by interacting our measure of HBW with the two following variables. First, following the UKHLS-derived measure of part-time schedule (*jbft_dv*), we classified women who work at most 30 hours per week as part-time workers. We interacted this variable with a simplified measure of HBW that collapsed regular and irregular home-based workers into a single category (home-based). This approach enabled us to maintain a sufficient sample size within each category of the interaction term. We thus distinguished the following five categories: (1) On-site and full-time; (2) Home-based and full-time; (3) On-site and part-time; (4) Home-based and part-time; and (5) Non-employed. This interaction between HBW and part-time work was performed only in the analysis of the transition to second child, as it is not common for childless women to reduce their working hours.

Second, we measured commuting time by way of women’s responses to the following question (*jbttwt*): *About how much time does it usually take for you to get to work each day, door to door (in minutes)?* This measure is available for employed women only. We included it into our models as a categorical variable distinguishing three levels: (1) 0–20 minutes; (2) 21–44 minutes; and (3) 45 minutes or more. We then interacted commuting time with our main measure of HBW, for the sample of employed women. For most of our employed respondents the commuting time was larger than zero, although 95 women reported zero commuting time. Importantly, women who participate in regular HBW may still have a non-zero commuting time, representing the duration of their commute when they work from their employer’s premises. Responses of zero were retained in our analysis, but we also performed a robustness check to verify whether and how they affected our findings (see Robustness checks subsection).

### Control variables

We included in our models a series of control variables: women’s age (18–24, 25–29, 30–34, 35–39, 40–44); ethnicity (British/Irish, Asian, Indian, Black, Other white, Other); partnership status (cohabiting, married); family orientation (as determined by the question *How important is the family to your sense of who you are?*, with categorical answers grouped into ‘very important’ vs ‘other’); educational level (classified following the UKHLS-derived measure of educational qualification into ‘high’, encompassing degree or higher, and ‘medium or low’, which includes A level, general secondary education, and below); income (*fimnnet_dv*: monthly total net personal income with no deductions, in quartiles); and time period (2009–12, 2013–16, 2017–19). We also added partners’ socio-economic characteristics, such as employment status (employed, self-employed, not working) and income (*fimnnet_dv*, in quartiles). The models for the transition to the second child additionally included age of the first child (0–1, 2–3, 4–6, 7+ years) and a dummy variable measuring the use of external childcare (*Do you ever use any type of (external) childcare for your child/-ren?*). Details on the control variables and summary statistics for all variables used in this analysis can be found in the supplementary material (Table A1).

### Method

We applied discrete-time, random-effects complementary log–log (cloglog) models separately for the transitions to first and second births. Random effects allowed us to account for the correlation between panel observations clustered for the individual (Hartzel et al. 2001), while cloglog specification is preferred over the logit or probit if the event is rare (Mills 2010). Our main explanatory variable, the moderating variables, and the control covariates were all lagged, as births occur after the decision to have a child is made. We lagged them by a year if the woman was not pregnant in the wave preceding childbearing or by two years if the woman was already pregnant in the wave before childbearing and this pregnancy had resulted in a live birth.

Most of the variables we included are measured annually, except the measure of HBW. These data are collected within the UKHLS Work Conditions module, which is included in the survey every second year, starting with Wave 2. We implemented the following strategy to impute these missing data. For waves in which working arrangements were not collected, we imputed the missing value by using the answers from the next nearest wave that collected this information, but only if a woman had not changed her job or employer between these two waves (so-called ‘imputation up’*)*. If a woman had changed her job and/or employer, we imputed the missing value by linear bootstrapping (less than 5 per cent of all values were bootstrapped). As such, we assumed that flexible working arrangements had not changed if there was no change in job/employer. Similarly, data on family orientation are collected every third wave (Adult Identity module, Waves 2, 5, and 8). We imputed the missing data from the next/previous nearest non-missing value and bootstrapped the remaining missing cases. We checked the robustness of our results on this imputation strategy (for details see Robustness checks subsection).

### Selection

We acknowledge that the potential selection of women into HBW may have impacted our findings. First, highly educated women and women in high-status occupations are likely to be over-represented among those who work from home. These women may differ in their childbearing plans from less educated women or women with low-status occupations. To rule out the possibility that the selection of highly educated women or women holding high-status occupations into HBW affected our findings, we repeated our analyses on: (a) the sample of highly educated women; and (b) the sample of women holding high-status occupations. Second, women who are more inclined to have children may choose jobs that grant access to HBW and even start working from home in advance, with the intention of having a child in future. We accounted for this type of selection by controlling for women’s family orientation (as described earlier).

## Results

### Descriptive statistics

Before we discuss our findings on the relationship between HBW and birth transitions, we first provide some basic information about women in our sample who work from home in comparison to on-site workers. Past research has indicated that women who work from home are usually highly educated and work in occupations at the top of the occupational hierarchy (Ammons and Markham 2004; Chung and Van Der Horst 2020), although there might also be diversity among home-based workers, with some holding particularly low quality and precarious jobs (Webster 2016). To interpret our findings properly, it is thus important to understand whether the home-based workers in our sample belong largely to the first category or whether HBW may also imply precarious labour market situation.

The findings presented in Table 1 clearly illustrate that socio-economic status is higher for both irregular and regular home-based workers in our sample than among on-site workers and non-working women. Specifically, among childless women engaged in regular HBW, 81.6 per cent have attained a degree or higher, and among mothers this percentage reaches 71.5 per cent. In comparison, 67.2 per cent of childless women and 55.7 per cent of mothers who work on-site have achieved higher education qualifications. Moreover, women who work from home hold higher occupational positions than their on-site counterparts. Among regular home-based workers, 60.2 per cent of childless women and 57.2 per cent of mothers occupy professional or managerial positions, whereas these occupations are held by only 35.2 per cent of childless on-site workers and 29.3 per cent of on-site-working mothers. Additionally, women who regularly work from home tend to earn higher wages, irrespective of parenthood status. Their average net monthly income exceeds £2,000, whereas for on-site working women, it barely surpasses £1,500. In terms of age distribution, we do not observe many differences between homeworking and on-site working women, apart from women who work from home being slightly more concentrated around the older childbearing ages (30–44).

**Table 1:** Descriptive statistics for childless women and first-time mothers by perceived access to and use of home-based working, UK 2009–19

Variable	Childless women				First-time mothers			
	Perceived access to and use of HBW				Perceived access to and use of HBW			
	*On-site working*	*Irregular HBW*	*Regular HBW*	*Not working*	*On-site working*	*Irregular HBW*	*Regular HBW*	*Not working*
Age								
* 18–24*	15.7	10.0	6.8	25.3	6.6	2.4	2.4	18.6
* 25–29*	35.1	31.4	25.3	24.1	19.4	12.0	10.0	24.4
* 30–34*	25.2	31.8	34.3	18.7	28.7	37.6	23.5	24.1
* 35–39*	13.4	15.7	19.7	18.1	27.0	30.5	40.9	19.7
* 40–44*	10.6	11.1	13.9	13.8	18.3	17.5	23.2	13.2
* *Total	100.0	100.0	100.0	100.0	100.0	100.0	100.0	100.0
Educational level								
* High*	67.2	75.7	81.6	43.8	55.7	67.5	71.5	38.7
* Medium or low*	32.8	24.3	18.4	56.2	44.3	32.5	28.5	61.3
* *Total	100.0	100.0	100.0	100.0	100.0	100.0	100.0	100.0
Occupation (ISCO-88 1 digit)								
* Professionals and managers*	35.2	47.6	60.2	–	29.3	46.4	57.2	–
* Associate professionals and clerks*	38.6	46.8	34.7	–	39.3	46.7	38.9	–
* Other (ISCO-88 groups 5–9)*	26.2	5.6	5.1	–	31.4	6.9	3.9	–
* *Total	100.0	100.0	100.0	–	100.0	100.0	100.0	–
Income: mean (standard deviation)	1,503 (684)	1,847 (712)	2,139 (1,043)	–	1,373 (725)	1,837 (1,112)	2,027 (868)	–

*Note:* ISCO-88 refers to the third version of the International Standard Classification of Occupations.

*Source:* Authors’ calculations based on UKHLS data.

### Regression results

**Figure 1:**
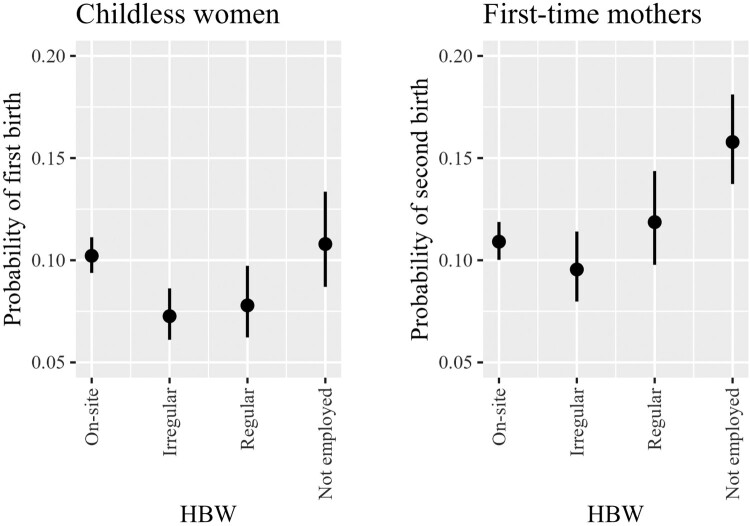
Predicted probabilities of first and second births by perceived access to and use of home-based working: all women, including the non-employed, UK 2009–19 *Notes*: Predicted probabilities and 83 per cent CIs are calculated based on the estimates from Model 1, which includes the HBW measure. Model 1 controls for woman’s age, period, ethnicity, partnership status, family orientation, educational level, income, and partner’s job status and income. The model for second births additionally includes age of the first child and use of external childcare. Models are based on women aged 18–44 living in a heterosexual union. *Source*: Authors’ calculations based on UKHLS data.

**Figure 2:**
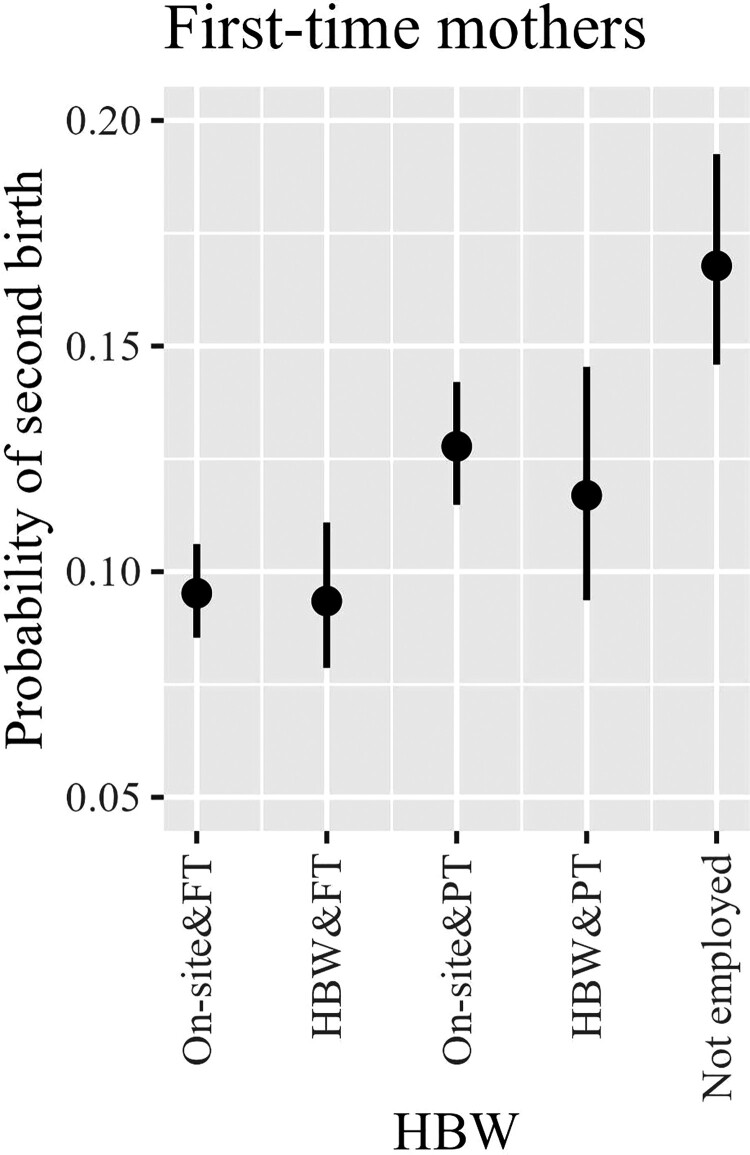
Predicted probabilities of second birth by home-based work and full/part-time schedules: all mothers, including the non-employed, UK 2009–19 *Notes*: Predicted probabilities and 83 per cent CIs are calculated based on the estimates from Model 2, which includes the simplified HBW measure (with regular and irregular HBW combined) interacted with full/part-time schedule. Model 2 controls for woman’s age, period, ethnicity, partnership status, family orientation, educational level, income, and partner’s job status and income, as well as age of the first child and use of external childcare. Models are based on women aged 18–44 living in a heterosexual union. *Source*: As for Figure 1.

**Figure 3:**
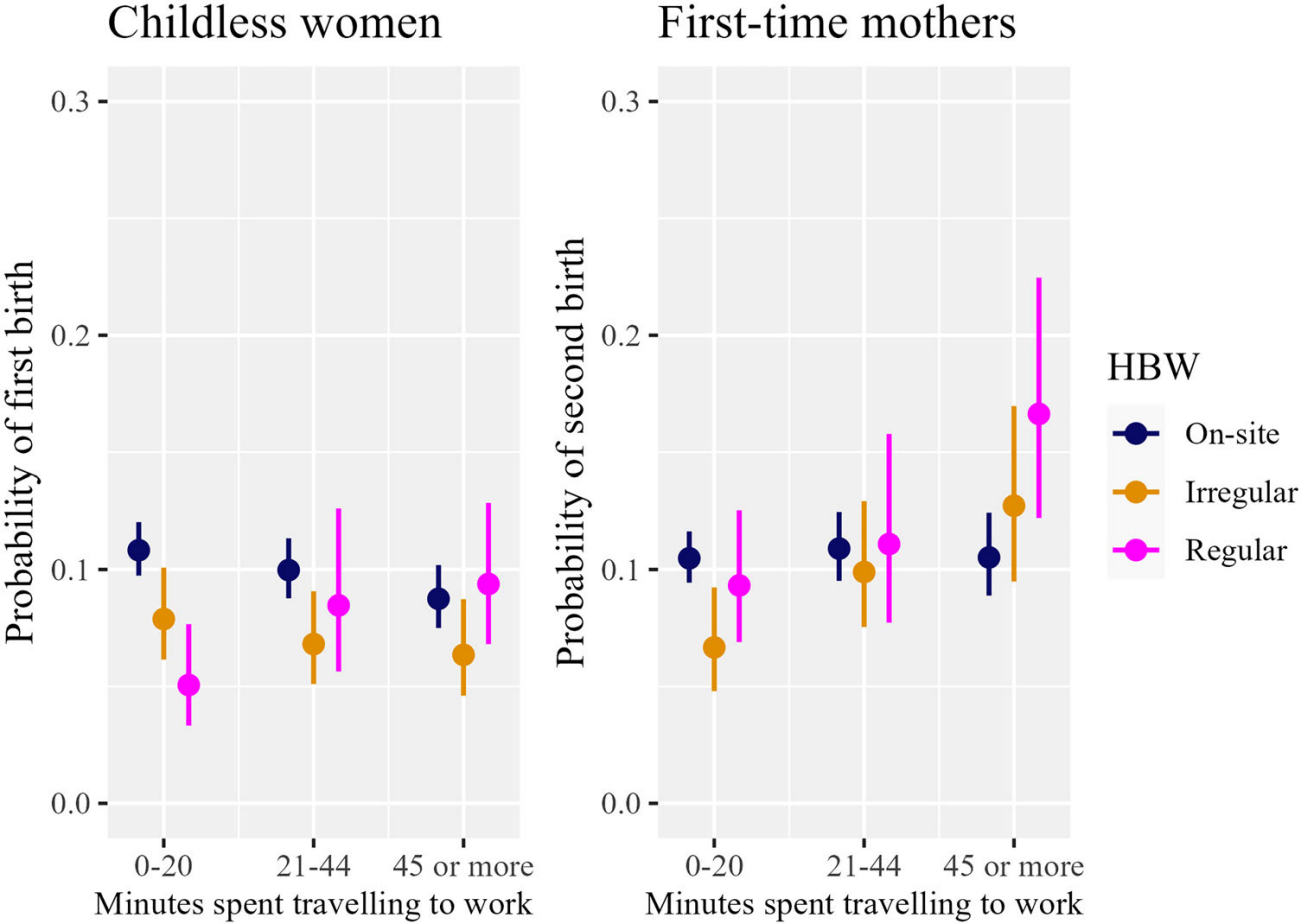
Predicted probabilities of first and second births by perceived access to and use of home-based working and by commuting time: employed women only, UK 2009–19 *Notes*: Predicted probabilities and 83 per cent CIs are calculated based on the estimates from Model 3 (employed women only), which includes the HBW measure interacted with commuting time. Model 3 controls for woman’s age, period, ethnicity, partnership status, family orientation, educational level, income, and partner’s job status and income. The model for second births additionally includes age of the first child and use of external childcare. Models are based on women aged 18–44 living in a heterosexual union. This figure is best viewed in colour online. *Source*: As for Figure 1.

We start our presentation of the analysis with a basic model that includes only our key explanatory variable (HBW) and controls (Model 1, Table 2). This model allows us to assess the main relationship between HBW and birth risk among all women, employed and non-employed. Next, based on the sample of mothers, we examine the role of a part-time schedule in the link between HBW and childbearing by introducing a new categorical measure that captures the interaction between HBW and part-time work (Model 2, Table 2). Finally, we investigate the role of commuting time by interacting it with our HBW measure (Model 3, Table 2), on a sample of employed women only, separately for childless women and mothers. For the sake of simplicity, we base our interpretations on predicted birth probabilities (plotted in Figures 1–3). We evaluate whether the difference between two predicted probabilities is significant by comparing 83 per cent confidence intervals (CIs). We do this following Austin and Hux (2002), who showed that two means differ from each other with the *p*-value at around 0.05 if 83 per cent CIs do not overlap.

**Table 2:** Estimates of the random-effects cloglog model on the probability of first and second births, UK 2009–19

	First births				Second births					
	Model 1		Model 3 (employed women only)		Model 1		Model 2		Model 3 (employed women only)	
*Predictors*	*RR*	*p-value*	*RR*	*p-value*	*RR*	*p-value*	*RR*	*p-value*	*RR*	*p-value*
Perceived access to and use of HBW *(ref. On-site working)*										
* Irregular HBW*	0.700	0.005	0.717	0.083	0.869	0.291	–	–	0.623	0.057
* Regular HBW*	0.752	0.091	0.453	0.012	1.093	0.548	–	–	0.884	0.595
* Not employed*	1.060	0.732	–	–	1.487	0.002	–	–	–	–
Home-based work and Full/part-time schedule (ref. *On-site and Full-time*)										
* HBW and Full-time*	–	–	–	–	–	–	0.981	0.882	–	–
* On-site and Part-time*	–	–	–	–	–	–	1.366	0.002	–	–
* HBW and Part-time*	–	–	–	–	–	–	1.243	0.218	–	–
* Not employed*	–	–	–	–	–	–	1.835	<0.001	–	–
Commuting time (ref. *0–20 minutes*)										
* 21–44 minutes*	–	–	0.917	0.419	–	–	–	–	1.042	0.704
* 45 minutes or more*	–	–	0.799	0.071	–	–	–	–	1.004	0.978
Home-based work and Commuting time										
* Irregular and 21–44 minutes*	–	–	0.937	0.825	–	–	–	–	1.448	0.257
* Regular and 21–44 minutes*	–	–	1.858	0.159	–	–	–	–	1.153	0.692
* Irregular and 45 minutes or more*	–	–	1.000	0.999	–	–	–	–	1.965	0.053
* Regular and 45 minutes or more*	–	–	2.374	0.032	–	–	–	–	1.853	0.076
Intercept	0.276	<0.001	0.294	<0.001	0.192	<0.001	0.152	<0.001	0.201	<0.001
Age (ref. *30–34*)										
* 18–24*	1.008	0.954	0.785	0.122	0.981	0.888	0.997	0.981	1.154	0.414
* 25–29*	0.880	0.194	0.836	0.079	1.084	0.381	1.096	0.323	1.040	0.716
* 35–39*	0.505	<0.001	0.501	<0.001	0.646	<0.001	0.651	<0.001	0.642	<0.001
* 40–44*	0.057	<0.001	0.048	<0.001	0.162	<0.001	0.162	<0.001	0.158	<0.001
Period (ref. *2009–12*)										
* 2013–16*	0.920	0.332	0.943	0.518	0.982	0.819	0.973	0.729	0.945	0.524
* 2017–19*	0.678	0.004	0.642	0.002	0.542	<0.001	0.532	<0.001	0.519	<0.001
Ethnicity (ref. *British/Irish*)										
* Asian*	0.590	0.051	0.676	0.163	0.947	0.780	0.963	0.844	1.210	0.436
* Indian*	0.657	0.014	0.775	0.199	0.758	0.037	0.777	0.057	0.761	0.135
* Black*	0.829	0.524	0.863	0.631	1.349	0.155	1.437	0.085	1.421	0.152
* Other white*	0.855	0.412	0.829	0.363	0.536	0.001	0.558	0.002	0.554	0.008
* Other*	0.424	0.080	0.620	0.338	2.157	0.015	2.134	0.016	2.561	0.018
Cohabiting (*ref. Married*)		<0.001	0.359	<0.001	0.828	0.035	0.823	0.029	0.805	0.037
Family orientation	1.387	0.001	1.381	0.002	1.123	0.261	1.125	0.254	1.116	0.368
Higher education	0.990	0.914	1.007	0.946	1.161	0.066	1.167	0.057	1.163	0.116
Income (*ref. Bottom quartile)*										
* Quartile 2*	0.712	0.009	0.710	0.011	1.123	0.339	1.149	0.252	1.199	0.258
* Quartile 3*	1.184	0.191	1.151	0.292	1.090	0.509	1.241	0.111	1.156	0.371
* Top quartile*	1.382	0.021	1.286	0.084	1.214	0.154	1.480	0.008	1.284	0.139
Partner’s job status (*ref. Employed)*										
* Self-employed*	1.087	0.539	1.143	0.347	1.171	0.139	1.170	0.142	1.200	0.148
* Not working*	0.670	0.041	0.546	0.018	1.253	0.153	1.237	0.178	1.315	0.243
Partner’s income (*ref. Bottom quartile)*										
* Quartile 2*	1.020	0.870	1.098	0.463	1.052	0.631	1.059	0.583	1.005	0.970
* Quartile 3*	0.862	0.235	0.922	0.546	0.938	0.570	0.940	0.578	0.871	0.283
* Top quartile*	0.956	0.724	1.086	0.542	1.101	0.391	1.086	0.460	1.005	0.968
Use of external childcare	–	–	–	–	1.479	<0.001	1.442	<0.001	1.542	<0.001
First child’s age (ref. *2–3*)										
* 0–1*	–	–	–	–	0.848	0.067	0.858	0.089	0.762	0.009
* 4–6*	–	–	–	–	0.783	0.026	0.783	0.026	0.719	0.009
* 7 or above*	–	–	–	–	0.282	<0.001	0.287	<0.001	0.229	<0.001
*N (number of women)*	2,095		1,914		2,164		2,164		1,733	
Observations (woman-years)	5,703		5,210		5,329		5,329		4,233	

*Source:* As for Table 1.

#### HBW and birth risks

In the first step, we investigate whether HBW is related to fertility transitions in our sample of women (Model 1, Table 2). We find that both categories of home-based workers (occasional and regular) are less likely to have a first child than women who work on-site or are not employed (Figure 1, left-hand side). As such, even after controlling for socio-economic status (educational level and income), we find that childless women who have the opportunity to work from home tend to postpone or abandon their transition to motherhood compared with other women, namely those working on-site as well as the non-employed. Regarding mothers, home-based workers (either regular or irregular) do not differ significantly in their likelihood to have a second child from on-site working women (Figure 1, right-hand side). However, comparing different categories of working women with those who do not work for pay yields interesting results. While on-site working women are less likely to progress to a second child than women who are not employed, women regularly working from home are as likely to have a second child as non-employed mothers (Figure 1, right-hand side). This finding needs to be corroborated in future research, as clearly the CI around the estimated second-birth probability of women working regularly from home is relatively large because the number of regular home-based workers is small. Nonetheless, it may indicate that in contrast to on-site work, HBW has the potential to reduce work–family conflict, with the result that mothers working from home are as likely to have a second child as those who do not work.

#### Part-time job

Next, in Model 2, we examine whether the birth risks of mothers working on-site vs mothers working from home (on a regular or irregular basis) depend on their work schedule (full-/part-time). Contrary to our expectations, we do not find that HBW particularly supports childbearing among full-time working mothers. In fact, no substantial differences are observed between mothers working full-time on-site vs mothers working full-time from home (Figure 2). Notably, we find higher second-birth probabilities for women working part-time on-site than for full-time working women, regardless of whether the latter worked on-site or from home. Interestingly, we observe a slightly elevated likelihood of a second birth among women involved in part-time home-based work. However, the increase is statistically insignificant, possibly due to small sample sizes leading to a wider CI, emphasizing the need for validation in future research.

#### Commuting time

Finally, in Model 3, we assess the role of commuting time (time taken to travel the distance between home and workplace) and find that HBW facilitates birth transitions for women with long commutes. More specifically, women who work from home regularly and live near their workplaces (0–20 minute commute each way) are less likely to have a first child than those who go out to work (Figure 3, left-hand side). This finding remains consistent regardless of women’s health status (see Robustness checks subsection). However, the probability of a first birth among women who regularly work from home increases with rising commuting time and becomes similar to that of on-site workers once commuting time exceeds 20 minutes. As such, the benefits from regular HBW where there is a long commute compensate for drawbacks that this working arrangement brings to childless women. The gains from not needing to commute are even more evident among mothers: those who can work from home regularly and gain a substantial amount of time (45 minutes or more) thanks to not commuting are more likely to have a second child than those who go out to work (Figure 3, right-hand side). That is not the case with short commuting times.

### Robustness checks

We considered several additional models (results available on request) in order to verify the robustness of our results and potential selection mechanisms. First, to account for the possibility that women who reported zero commuting time impacted our finding, we excluded them from our sample of women and re-evaluated our models. This did not lead to a significant change in our findings. Second, we considered the variable *Main job location: at home* as an additional measure of HBW, adding it to our basic models alongside our standard HBW measure. The findings did not change significantly. Third, we included health status in our models to ensure that the observed negative association between HBW and birth risk for women with a short commute was not influenced by health conditions. This adjustment did not modify our findings. Similarly, the inclusion of occupation into the models conducted on a sample of employed women did not alter our findings.

Further, we made several imputations of the missing data on the HBW measure in order to assess how much the imputed values impacted our results. We allowed for different imputation strategies (bootstrapping, imputation up and down), and the results held. Finally, we verified whether our findings were affected by the over-representation of women with little access to HBW in the reference category (on-site workers). As the eligibility to work from home is higher for high-status occupations, our results may reflect the selection of women with certain fertility plans into high occupational positions rather than the actual effect of HBW on childbearing. To this end, we repeated our analysis on three subsamples: (1) women holding the highest occupational positions (International Standard Classification of Occupations [ISCO] one-digit codes 1–4); (2) women performing jobs that (according to Dingel and Neiman 2020) can be done from home; and (3) women with higher education only. We reran all our models on these subsamples and obtained very similar findings to those run on full samples. The results obtained from the first subsample are included in the supplementary material (Figures A1–A3). The remaining results (subsamples 2 and 3) and the results of all other additional analyses presented in this subsection are available on request.

## Discussion and conclusions

In their seminal paper on fertility and women’s employment, Brewster and Rindfuss (2000) argued that incompatibilities between paid work and care are a typical characteristic of industrialized societies, where workplaces are often situated far from homes and work schedules and childcare needs are not easily reconciled. They claimed that this physical separation of paid work and family life was responsible for the dramatic decline in fertility that took place in Western Europe, North America, and Australia in the second half of the twentieth century as large numbers of women entered the labour force. A mere two decades after the publication of their study, it has been estimated that around 40 per cent of jobs in Western Europe and the United States can be performed entirely from home (Dingel and Neiman 2020). In light of this estimate, a question has emerged about the consequences of the possible spread of HBW for fertility. Surprisingly, although plenty of research has investigated the link between HBW and various aspects of workers’ lives, no study has yet examined its relation to childbearing. In this paper, we have closed this gap by examining, in the pre-pandemic context of the UK (2009–19), whether women who had access to HBW or did at least some of their paid work from home were more likely to have a(nother) child.

We showed that childless women who worked from home—regularly or irregularly—were *less* likely to enter motherhood than childless women working on-site or not employed. This finding is in line with the strand of literature showing that HBW has negative consequences for women’s professional careers, including lower wages and less chance of being promoted compared with their counterparts with similar tenure, position, and experience but who work on-site (Munsch 2016; Golden and Eddleston 2020; Kasperska et al. 2023). Childless women who work from home will therefore face higher opportunity costs of parenting. As a result, in our analysis they proved to be the group that was most likely to postpone childbearing, presumably in order to establish their position in the labour market before becoming mothers.

Further, we showed that HBW was unrelated to the progression to a second child. Like childless women, mothers working from home not only have fewer opportunities for promotion (Munsch 2016) but must also multitask more and shoulder a larger load of unpaid labour (Ammons and Markham 2004; Kurowska 2020). However, these negative consequences of HBW are likely offset by the advantages of this working arrangement. These include greater flexibility in organizing work around family obligations (Chung and Van der Lippe 2020), time saved thanks to not having to commute, and the ability to perform paid work in parallel to household tasks (Hill et al. 2003; Bai et al. 2021), all of which help mothers to combine paid work with care and may encourage them to have another child. It was also notable from our analysis that full-time working mothers did not display a higher second-birth probability when they worked from home. This finding stands in contrast to our expectation that HBW would be especially helpful to full-time working mothers, who face the most burdensome time constraints. However, we did find that part-time work was positively linked to second-birth risks among on-site working mothers. This finding suggests that, in the pre-pandemic context, reduced working time was the arrangement most conducive to subsequent childbearing. Future studies should investigate whether this finding still holds after the pandemic.

All in all, our findings do not support the idea that an increase in paid work at home will lead to an immediate increase in fertility. Instead, we found that HBW may provide advantages primarily for a specific group of women: those who live far from their workplace and thus would have to spend a great deal of time commuting if they worked on-site. For larger gains in total fertility to be achieved, HBW would need to entail lower costs for the remaining women. These costs include the higher expectations placed on homeworking women to perform more housework and childcare (Ammons and Markham 2004) and thus to shoulder a higher total workload (Kurowska 2020). The costs may also extend to psychological distress resulting from multitasking or fragmented working time (Hill et al. 2003). Last but not least, HBW may also entail negative consequences for women’s careers, and these will need to be eliminated if HBW is going to be a successful means for combining paid work and care. These negative consequences of HBW on women’s careers often lead to female home-based workers being perceived negatively by employers, who associate this working arrangement with a lower commitment to work and a strong attachment to care obligations (Munsch 2016; Kasperska et al. 2023). In the context of persisting negative consequences of HBW on women’s careers, it is unlikely that the recently observed downward trend in first-birth rates in the UK will decelerate (Berrington 2017; Ermisch 2021). This is particularly salient when we take into account our research results, which indicated a notable association between HBW and postponement of the transition to motherhood.

It stands to reason that the spread of HBW will not have spectacularly positive effects on fertility without further progress in gender equality and higher acceptance of flexible working arrangements among employers. During the recent Covid-19 pandemic, the widespread experience of homeworking, which affected nearly half of the UK workforce (Mutebi and Hobbs 2022), may have accelerated the process toward higher acceptance of HBW. This increased prevalence of HBW has the potential to reduce the stigma associated with flexibility. Before the pandemic, employers often feared a decline in productivity among employees working from home. However, this fear was not realized during the pandemic. In fact, a survey conducted by the Chartered Institute of Personnel and Development (CIPD) in Autumn 2021 revealed that 41 per cent of employers reported increased productivity or efficiency as a result of HBW, while only 18 per cent reported a decrease (CIPD 2022). Similarly, an analysis by the Office for National Statistics (ONS) in early 2022 found that 24 per cent of businesses were either currently using or planning to use HBW as a permanent business model. Furthermore, two-fifths of these businesses attributed their decision to the increase in productivity (ONS 2022).

However, the Covid-19 pandemic has exposed and exacerbated many gendered inequalities in the labour market. For example, women faced higher levels of workplace inequality and discrimination than men during the pandemic (Foley and Cooper 2021), leading to a greater risk of job loss (Flor et al. 2022). Moreover, while working from home, women experienced disproportionate disruption to their paid work due to higher responsibilities for childcare and housework (Carli 2020). These added pressures also contributed to higher pandemic-related psychological stress among women than men (Wade et al. 2021). In summary, while widespread homeworking has shown potential for increased productivity and efficiency, thereby lowering flexibility stigma, the persistence of gender inequalities in the labour market may hinder the use of this mode of working among women. It is thus important to address gender inequalities and promote flexible working arrangements for both men and women in order to realize the positive effects of HBW on fertility rates fully.

Our study was not without limitations. First, our data—one of the few panel surveys to provide longitudinal information on workplace flexibility—did not allow us to distinguish precisely between access to HBW and its use. We were able to separate those who used HBW on a regular basis from those who had access to HBW but did not use it regularly. But within the latter group, we could not distinguish those workers who did not use HBW at all. Moreover, it remains unclear what ‘regular use’ means to respondents and how frequently this might imply HBW is used. As such, those designing future surveys should look carefully at how questions on HBW are worded, particularly as teleworking will certainly be more widespread during this decade than it was in the years covered by this study.

A related issue is whether all survey respondents who answered the question on having access to working from home in their job indeed knew with certainty that they had such an option. This problem should be less acute in future, as the Covid-19 pandemic made it much more evident to people which jobs and occupations could be done from home and what employers’ attitudes were to this working arrangement. We set out to minimize this problem by locating our study in the UK, which provides every employee with the right to ask for flexible working, leading to its population reporting one of the highest shares of HBW among developed countries. Nonetheless, the number of women who regularly work from home was quite low, which might have affected the significance of some of our estimates.

Nor can we rule out the possibility that women who were able to work from home before the pandemic had specific unobserved characteristics related to childbearing that led them to HBW. The sensitivity analyses we performed did not provide evidence for a selection of women into HBW according to whether they were family oriented or not. Nevertheless, we do acknowledge that selection into employment may be essential for the link between HBW and childbearing, as women who left the labour market in order to fulfil their childbearing plans might have stayed in the labour force if HBW had been available to them. We were not able to capture that fully in our study. Furthermore, some women may choose not to have a child, irrespective of their flexible working arrangements. If there were a third factor that influenced both the desire for childlessness and the utilization of HBW, we might have observed a spurious negative relationship between HBW and entry to motherhood. That could occur, for instance, if some women prioritize a convenient lifestyle with a high balance between their paid work and personal life but without the intention of having children. To gain a better understanding of the observed relationship, it would have been beneficial to focus exclusively on women who genuinely intended to have a child. Regrettably, we were unable to conduct this analysis due to the absence of information on women’s fertility intentions in our study. More research should be conducted on this issue, particularly in the post-pandemic context, which is characterized by a more widespread and less selective prevalence of HBW.

Despite these limitations, our study has made an important contribution to research in the field of family and work by being the first comprehensive study on HBW and fertility. This paper not only provides novel empirical findings but also outlines a theoretical framework on how HBW may affect fertility behaviours. As such, the study has potential to stimulate future research on the topic, which will likely become widely discussed among demographers, thanks to the rapid development of information and communication technologies. These technologies were conducive to the use of HBW during the Covid-19 pandemic and the change in both employers’ and employees’ behaviours and attitudes in that respect. Future studies could use better analytical methods to address the selection bias or to isolate tempo from quantum effects. More cross-national comparative research is also needed to examine whether our findings hold in other welfare state contexts as well as in different gender or care regimes.

## Supplementary Material

Supplemental Material
