# Supplementary material for: Home-based work and childbearing
Source: Popul Stud (Camb). 2024 Feb 6;78(3):525–45. doi: 10.1080/00324728.2023.2287510 (PMC11493054; doi:10.1080/00324728.2023.2287510)
Supplement: Supplemental Material [file RPST_A_2287510_SM6296.pdf]

TABLE A1. Summary statistics - categorical covariates

Variable	Levels	CHILDLESS Frequency	CHILDLESS Percent	MOTHERS Frequency	MOTHERS Percent
Perceived access to and use of HBW	On-site working	4033	70.8	3435	64.4
	Irregular HBW	781	13.7	458	8.6
	Regular HBW	396	6.9	340	6.4
	Not employed	493	8.6	1096	20.6
Home-based work and Full/part-time schedule	On-site and Full-time			2009	37.7
	HBW and Full-time			562	10.5
	On-site and Part-time			1426	26.8
	HBW and Part-time			236	4.4
	Not employed			1096	20.6
Home-based work and Commuting time	On-site and 0–20 min	1995	38.3	2093	49.4
	Irregular and 0–20 min	313	6.0	179	4.2
	Regular and 0–20 min	147	2.8	171	4.0
	On-site and 21–44 min	1190	22.8	858	20.3
	Irregular and 21–44 min	253	4.9	167	3.9
	Regular and 21–44 min	107	2.1	85	2.0
	On-site and 45 min or more	848	16.3	484	11.4
	Irregular and 45 min or more	215	4.1	112	2.6
	Regular and 45 min or more	142	2.7	84	2.0
Age	18–24	865	15.2	450	8.4
	25–29	1875	32.8	1024	19.2
	30–34	1493	26.2	1502	28.2
	35–39	832	14.6	1421	26.7
	40–44	638	11.2	932	17.5
Period	2009–12	2429	42.6	2315	43.4
	2013–16	2468	43.3	2279	42.8
	2017–19	806	14.1	735	13.8
Ethnicity	British/Irish	4690	82.2	4099	76.9
	Asian	173	3.0	187	3.5
	Indian	380	6.7	547	10.3
	Black	112	2.0	123	2.3
	Other white	280	4.9	337	6.3
	Other	68	1.2	36	0.7
Cohabiting	married	2748	48.2	3797	71.3
	cohabiting	2955	51.8	1532	28.7
Family oriented	no	1490	26.1	810	15.2
	yes	4213	73.9	4519	84.8
Educational level	medium or low	1863	32.7	2438	45.7
	high	3840	67.3	2891	54.3
Income	Bottom 25	1412	24.8	1281	24.0
	25–50	1433	25.1	1363	25.6
	50–75	1434	25.1	1334	25.0
	Top 25	1424	25.0	1351	25.4
Partner's job status	Employed	4712	82.6	4300	80.7
	Self-employed	566	9.9	672	12.6
	Not working	425	7.5	357	6.7
Partner's income	Bottom 25	1470	25.8	1324	24.8
	25–50	1378	24.2	1363	25.6

	50–75	1435	25.1	1307	24.5
	Top 25	1420	24.9	1335	25.1
First child's age	0–1			1913	35.9
	2–3			1142	21.4
	4–6			906	17.0
	7 or above			1368	25.7
Use of external childcare	no			2546	47.8
	yes			2783	52.2
	Total	5307	100.0	5329	100.0

Source: Authors' calculations based on UKHLS data

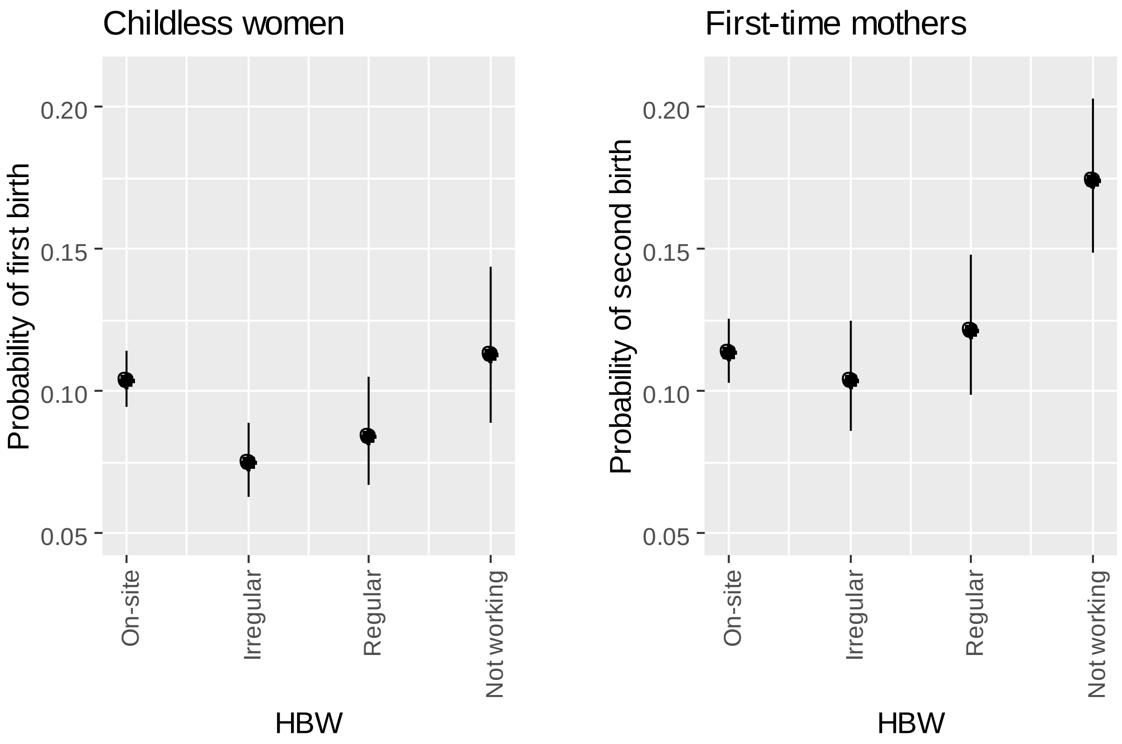

Figure A1. Predicted probabilities of first and second births by perceived access to and use of home-based working: women holding 1 to 4 ISCO-88 occupations and the non-employed women, UK 2009-2019

Source: Authors’ calculations based on UKHLS data.

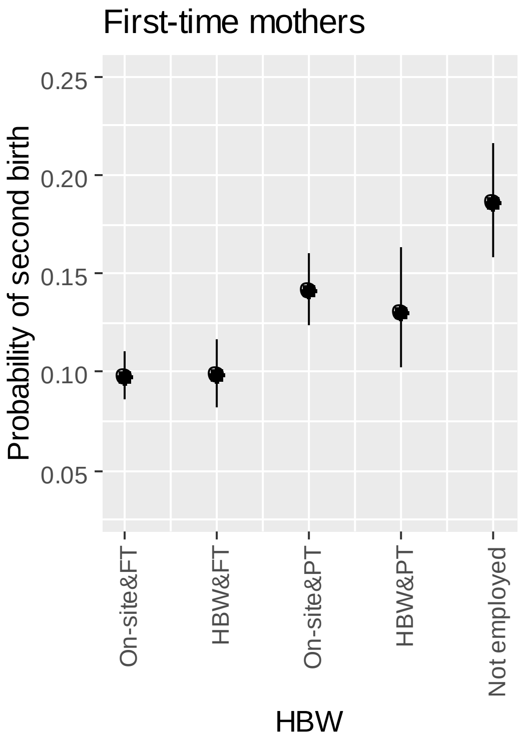

Figure A2. Predicted probabilities of second birth by home-based work and full/part-time schedules: women holding 1 to 4 ISCO-88 occupations and the non-employed women, UK 2009-19

Source: As for Figure A1.

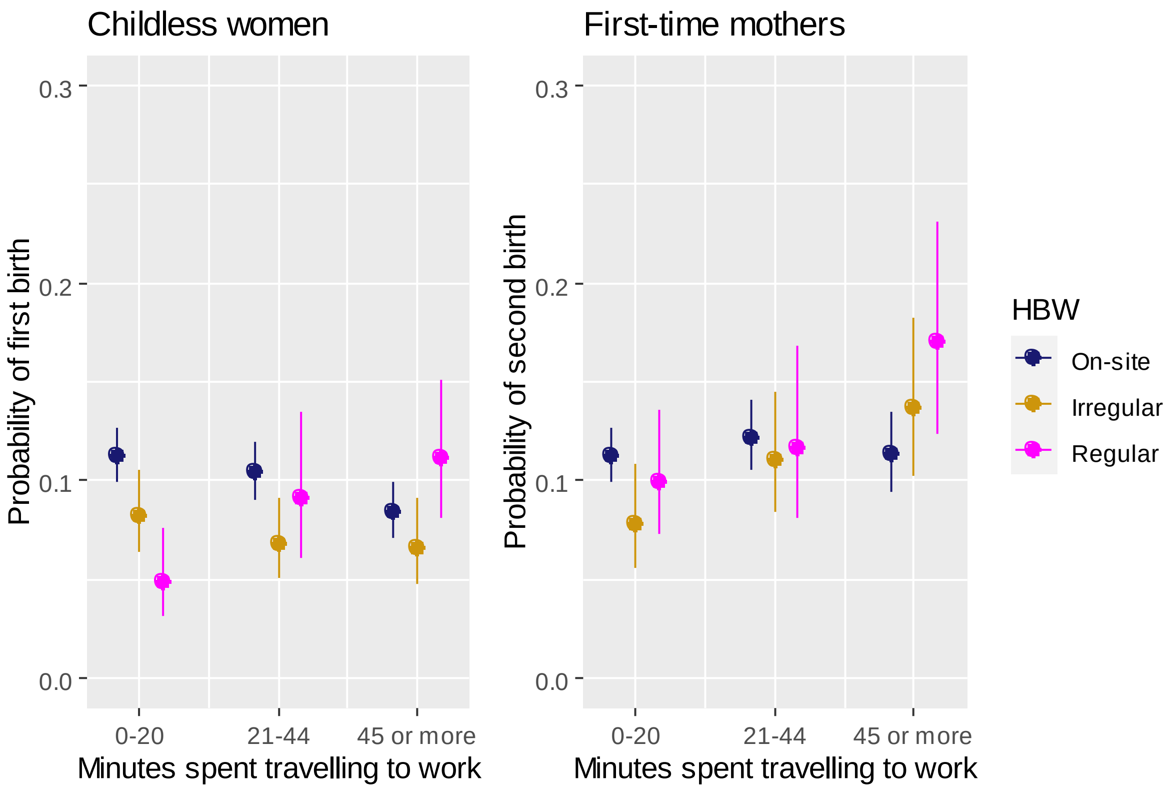

Figure A3. Predicted probabilities of first and second births by perceived access to and use of home-based working and by commuting time: employed women holding 1 to 4 ISCO-99 occupations, UK 2009-19

Source: As for Figure A1.
